# A Novel Image‐Based Assessment for Pork Quality Classification via Electrochemical Impedance Combined With Sensory Evaluation

**DOI:** 10.1002/fsn3.4600

**Published:** 2024-12-10

**Authors:** Yuezhong Mao, Chuanyong Yan, Xiaotong Liu, Shuangni Shi, Yumei Qin, Shiyi Tian

**Affiliations:** ^1^ School of Food Science and Biotechnology Zhejiang Gongshang University Zhejiang China; ^2^ Food Nutrition Science Centre, School of Food Science and Biotechnology Zhejiang Gongshang University Hangzhou China

**Keywords:** electrochemical impedance, image‐based assessment, pork quality, sensory evaluation

## Abstract

Rapid detection of pork quality has garnered increasing attention due to its status as one of the most widely consumed meats in the world. This study developed an electrochemical impedance combined with sensory evaluation method to achieve real‐time imaging and quality assessment of pork. The optimal parameters for pork detection were determined through system performance tests and a Design of Experiment, which included the use of an adjacent excitation pattern, an excitation current of 15 mA at 10 kHz, a detector diameter of 5 cm, and stainless‐steel electrodes embedded in the pork. This method facilitated real‐time imaging, evaluated the distribution of lean and fat, and accurately distinguished different qualities, demonstrating strong correlation with sensory assessments. Furthermore, it could assess pork with varying levels of freshness, achieving a distinguishing index of 99.93. Hence, this approach holds promising potential for applications in the domain of meat quality assessment.

## Graphical abstract

This study developed an electrochemical impedance combined with sensory evaluation method to achieve real‐time imaging and quality assessment of pork. This method facilitated real‐time imaging, evaluated the distribution of lean and fat, and accurately distinguished different qualities, demonstrating strong correlation with sensory assessments. Furthermore, it could assess pork with varying levels of freshness, achieving a distinguishing index of 99.93.

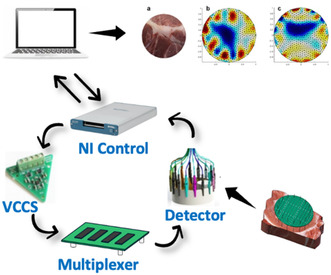

## 1 Introduction

Pork played a crucial role in the daily diet of people worldwide, and its quality directly impacted consumer health and safety (Godziszewska et al. 2017; Baer, Miller, and Dilger 2013). Quality inspection of pork not only ensured its freshness and freedom from contamination but also effectively detected diseases or drug residues, thereby safeguarding food quality and public health (Stahl et al. 2015; Sanchez et al. 2022). Strict enforcement of quality inspection could promote sustainable development in the pork industry, bolster consumer confidence, and drive a healthy market cycle. Hence, the significance of pork and its quality inspection was indisputable.

In terms of current technology, pork quality inspection techniques mainly consist of two methods. The first method involved artificial sensory evaluation by trained assessors. This intuitive and fundamental assessment relied primarily on human vision, olfaction, and touch (Guo et al. 2024; Zhao et al. 2024). Specifically, it entailed visually assessing the color and texture of pork for brightness and clarity; using olfaction to detect freshness or any off‐odors in the pork; and employing tactile sensation to evaluate elasticity and viscosity (Bassey et al. 2022; Barbin et al. 2012; Mao et al. 2024; Wei et al. 2024). This method was rapid and straightforward, enabling effective initial judgment of pork quality without specialized equipment‐a crucial means to ensure food safety and protect consumer rights. However, for a more comprehensive understanding of pork quality, artificial sensory evaluation often needed to be complemented with modern instrumental detection methods.

The second method for evaluating pork quality involved instrumental analysis, which utilized advanced equipment such as the Kjeldahl nitrogen analyzer, near‐infrared spectrometer (Barbin et al. 2013; Fan, Liao, and Cheng 2018; Zou et al. 2021), and gas chromatography‐mass spectrometry (Sun et al. 2018) to measure the moisture content, protein content, fat content, and volatile basic nitrogen (Chen et al. 2019; Gil et al. 2011) in pork. This approach allowed for simultaneous analysis of multiple components in pork, leading to a more comprehensive assessment of its quality. However, instrumental analysis required costly equipment, intricate operational procedures, and higher operator proficiency levels, thereby limiting its widespread application.

In recent years, owing to the continuous advancement of electrochemical technology, it found extensive application in meat inspection, particularly through the Electrical Impedance Tomography (EIT) method (Leitzke and Zangl 2020). EIT was a specialized electrochemical testing technique that utilized alternating current, or voltage applied to the boundary of the object under examination to generate cross‐sectional images (Darma and Takei 2021) reflecting impedance distribution (Zhao et al. 2017) or changes in the test area based on voltage or current distribution (Leng et al. 2019). However, the results obtained from EIT analysis were relatively autonomous and still might not have fully captured the true sensory attributes of pork as perceived by consumers.

It was evident that the current technology for pork quality detection had certain limitations and was not suitable for rapid analysis and early detection in industrial and commercial processing (Shi et al. 2021). Therefore, it was of great significance to explore and establish novel detection technologies that could meet these requirements for the development of the pork and other meat industries (Munekata et al. 2023).

This study aimed to employ sensory evaluation and EIT technology in establishing a detection system and method for rapid quality assessment of pork. First, a bioimpedance testing system was developed, and the sample imaging was accomplished using advanced algorithms. Second, the optimal detection conditions for applying the impedance imaging system to pork detection were determined through design of experiment (DOE). Finally, the established sensory evaluation standards were optimized and combined with impedance testing to achieve rapid visual assessment of pork quality.

## 2 Materials and Methods

### 2.1 Samples

Pork samples were obtained from Zhejiang Yonghui Supermarket. Various quality grades were selected at random, with the skin removed to retain only the lean and fat tissues. In order to prevent excessive current flow outside the measurement area during impedance imaging, the sample diameter should be approximately 1 cm larger than the detector. As a result, the pork was cut into 11 cm and 6 cm squares, with the 6 × 6 cm sample being obtained from the 11 × 11 cm sample. The samples were placed in plastic discs and assigned random numbers to avoid psychological bias towards specific numbers. Prior to detection and human sensory evaluation, the pork samples were meticulously placed into glass bottles and subsequently sealed with 3 M film to ensure optimal preservation. Afterward, the sealed pork samples were stored at a controlled temperature of 4°C. During EIT test, the pork samples were kept in the tank of EIT system. And during the human sensory evaluation, the pork samples were kept in the white glass dishes.

### 2.2 EIT System

#### 2.2.1 Electrode

The needle‐shaped electrodes, each 3 cm in length, were designed and purchased from Tianjin Ada Hengsheng Technology Co. Ltd. The electrodes, made of silver, copper, and stainless steel with a purity of 99.99%, were evenly arranged and fixed onto a plastic disc to form the detector.

#### 2.2.2 Detection Procedure

The EIT system utilized the USB‐6251 multifunction data acquisition card (National Instruments, USA) as both the signal excitation source and data acquisition terminal. This card was capable of generating standard sine wave signals, which were then converted from voltage to current signals through a single operational amplifier VCCS circuit based on the Howland current source. This configuration ensured the stability of the output signal's amplitude and frequency. The constant current signal generated was applied to the excitation electrodes based on a specified excitation pattern. At any given time, the system necessitated simultaneous connection of two excitation electrodes and two measurement electrodes to the circuit. Therefore, a multiplexer was employed to achieve rapid switching between the excitation and measurement channels.

**FIGURE 1:**
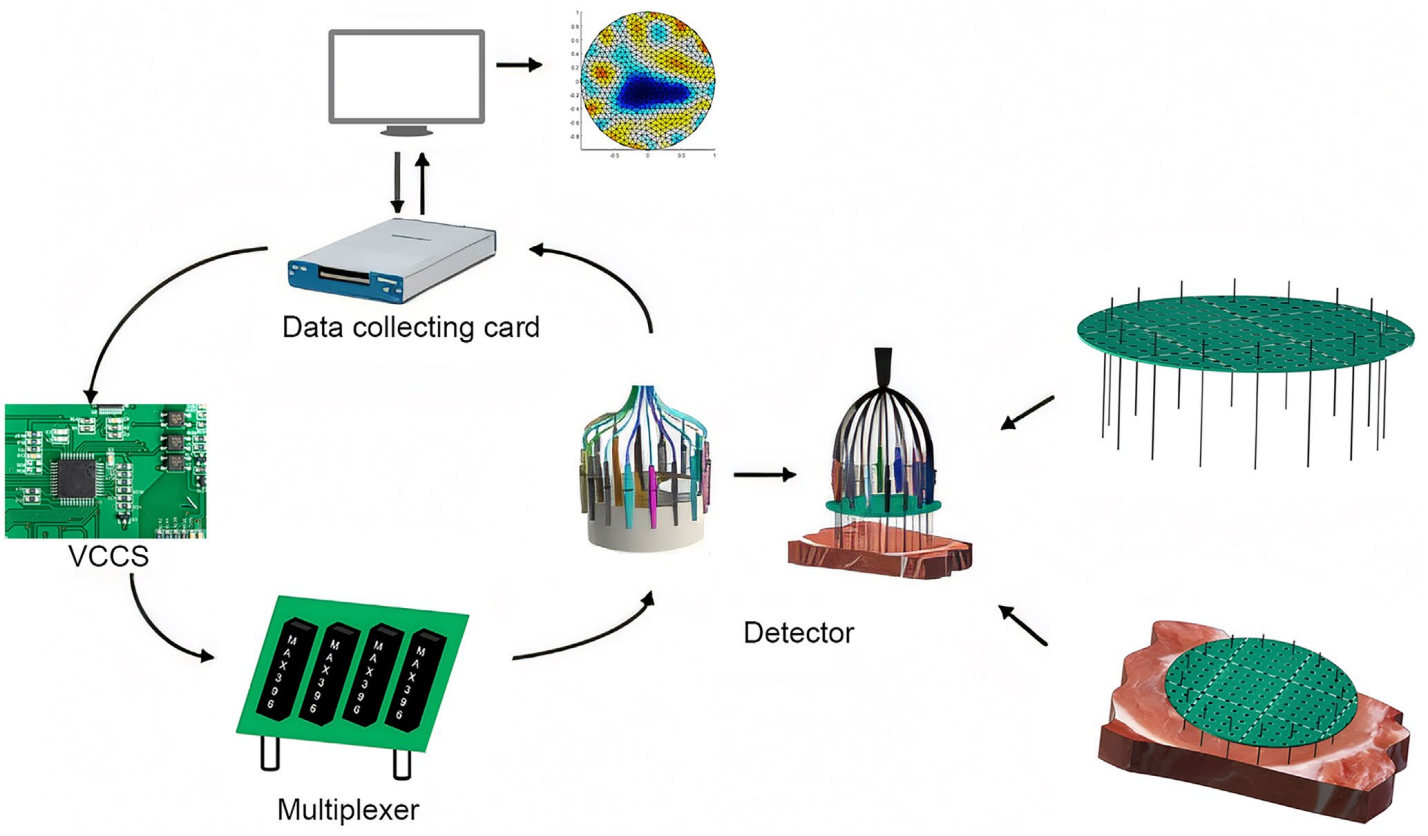
Electrochemical impedance detection system.

After injecting excitation current into the object under test, the boundary voltage signals were acquired using a data acquisition card. Subsequently, a demodulation algorithm was applied to calculate the real and imaginary components of the boundary voltage, as well as the amplitude and phase of the signal. The finite element method (FEM) was utilized to discretize the field of the object under test, with imaging functionality implemented jointly using MATLAB and the EIDORS software package. The finalized bioimpedance testing system and detection process are shown in Figure 1.

### 2.3 EIT System Performance Evaluation

#### 2.3.1 Hardware System Evaluation

Using a circular tank and a fixed resistor as the test subjects, the study assessed the channel consistency, signal‐to‐noise ratio (SNR), and system stability. The relative error of the maximum voltage measurement (*δ*_max_) was utilized to gauge the overall stability of the system.

**FIGURE 2:**
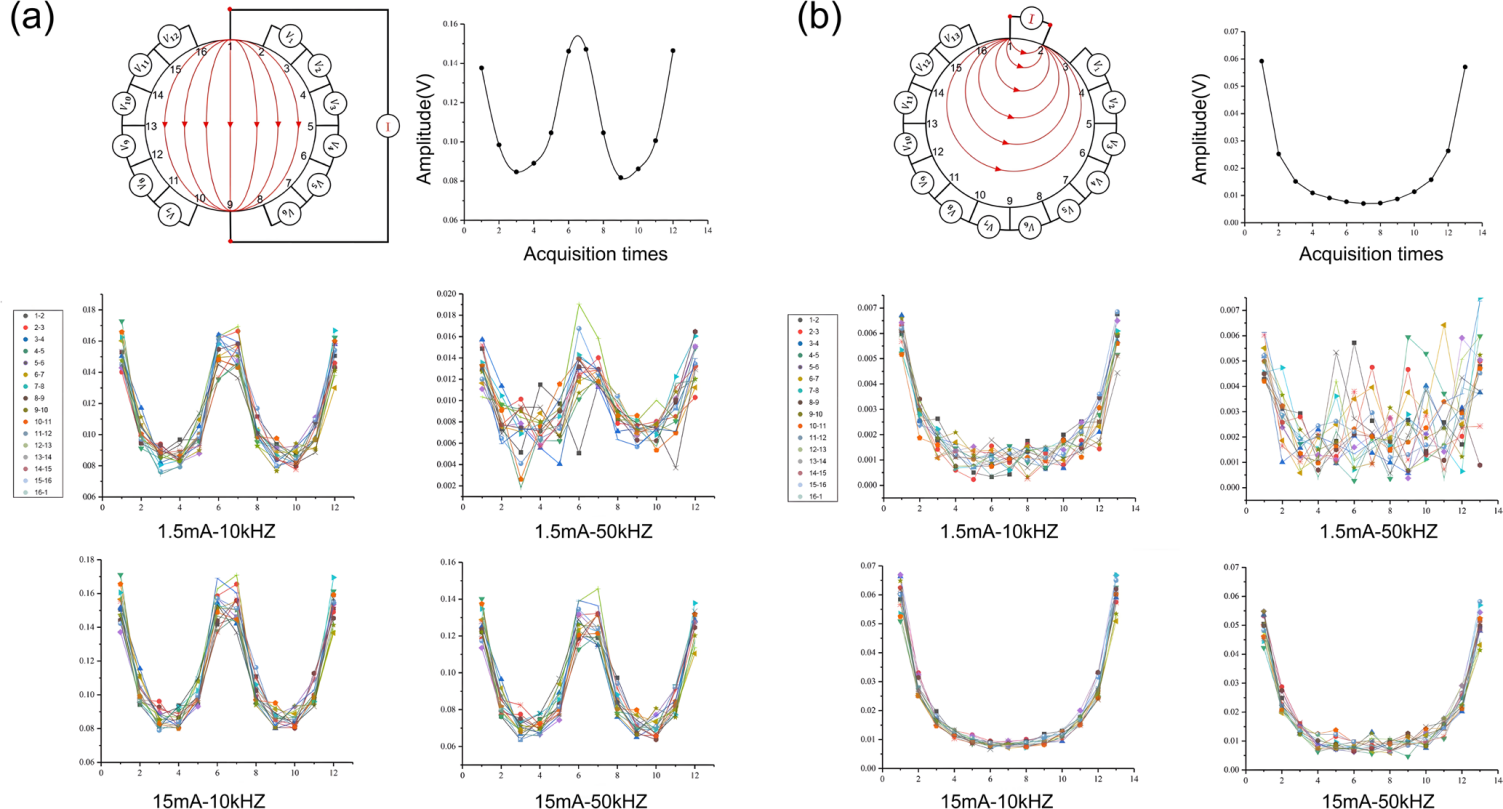
Channel consistency test for two excitation patterns: (a) opposite excitation pattern and (b) adjacent excitation pattern.

The tank, with a diameter of 10 cm and a height of 3 cm, was equipped with electrodes evenly distributed along its inner wall. A conductive medium of 0.9% NaCl solution was utilized within the tank, while the fixed resistor had a resistance value of 1 kΩ. The “foreign object,” a plastic cylindrical item produced from PLA material using advanced 3D printing techniques, was described in this paper. The specific experimental procedure is as detailed below:

1. Experiments were carried out using a stainless‐steel electrode detector in the absence of any foreign objects inside the tank. Both adjacent and opposite excitation patterns were utilized for detection, incorporating adjacent measurement pattern. The adjacent and opposite excitation patterns were as follows:

Excitation currents were sequentially applied to adjacent (or opposite) pairs of electrodes, and the potentials across neighboring electrode pairs, excluding those being excited, were alternately measured. This process continued until excitation currents had been applied to all adjacent (or opposite) electrode pairs. In the adjacent excitation pattern, excitation currents were sequentially applied to electrode pairs in the order of 1–2, 2–3, 3–4…, 15–16, and 16–1. In the opposite excitation pattern, excitation currents were applied to electrode pairs in the order of 1–9, 2–10, 3–11…, 15–7, and 16–8 (as shown in Figure 2).

2 The excitation current was conducted through the stationary resistor, and the voltage across the resistor was recorded at 5‐min intervals. This procedure was repeated 30 times.
3 Experiments were conducted under two distinct conditions: with and without the presence of foreign objects in the tank. The electrodes utilized in these tests consisted of silver, copper, or stainless steel. The voltage at the boundary of the tank was measured at intervals of 5 min, and this procedure was replicated 30 times to ensure consistent and reliable data.

The aforementioned tests were conducted using four distinct excitation currents: 1.5 mA‐10 kHz、1.5 mA‐50 kHz、15 mA‐10 kHz、15 mA–50 kHz.

#### 2.3.2 Image Quality Assessment

Image reconstruction was influenced by both the hardware system and imaging algorithms. In this section, imaging tests were primarily conducted under specific experimental conditions: stainless‐steel electrodes were used, an adjacent excitation pattern was employed, and the excitation current was set to 15 mA at a frequency of 10 kHz. To determine the optimal number of imaging units, different finite elements were divided, and foreign objects of varying sizes and numbers were placed in different positions for imaging, in order to evaluate the system's imaging performance.

1. A foreign object with a diameter of 35 mm and a height of 3 cm was placed between the 1 to 2 electrodes, and the measured field was divided into 126, 361, 633, 1108, and 1735 finite elements for image reconstruction.
2. The foreign object was placed in the middle of the 1–2 electrode, 5–6 electrode, 9–10 electrode, 13–14 electrode, and the center of the model for imaging.
3. Foreign objects of varying diameters (35 mm, 20 mm, 10 mm, and 7.5 mm) were positioned between 1 to 2 electrodes for imaging purposes.
4. One or more foreign objects with a diameter of 35 mm or 20 mm were positioned at different positions for imaging.

### 2.4 Optimization of EIT Experimental Parameters Based on DOE

#### 2.4.1 Design of Experiments (DOE)

DOE was a systematic methodology employed to identify which variables (or factors) exerted the greatest influence on a specific process. Its primary objective was to arrange experiments rationally, in order to obtain ideal experimental results and draw scientific conclusions.

Through preliminary experiments, significant influencing factors were identified, including excitation pattern, electrode spacing, electrode material, current magnitude, current frequency, and the size of foreign objects. The excitation patterns were classified into adjacent excitation and opposite excitation. The electrode spacings were set at 1.21 cm and 3.18 cm. The electrode materials used were stainless steel, copper, and silver. The current magnitudes were set at 15 mA and 1.5 mA. The current frequencies were set at 10 kHz and 50 kHz. The sizes of the foreign objects were categorized as no foreign object, foreign object with a diameter of 20 mm, and foreign object with a diameter of 35 mm.

A total factor design was created. According to the size of different foreign objects, 48 groups of standard‐order experimental design tables were generated by selecting a randomized experimental order. Experiments were then carried out, data were collected, and images were reconstructed. The quantitative index δ was used for evaluation. The experiment was repeated three times under each condition, and the average comparative value of the reconstructed image and the binarized model image was taken as the result. After obtaining the optimal parameters of the model, the experiment was designed and repeated three times to verify the reliability, based on the given optimization parameters.

#### 2.4.2 Image Assessment

The disparity in conductivity between the target and background was notable. As evident from the impedance imaging display method, the reconstructed image exhibited significant color differences between the foreign object and the background. Consequently, utilizing the binarized image after processing facilitated a more convenient inspection of the foreign object's position and size. Although the processed reconstructed image may have lost some information to a certain extent, its overall impact on the analysis remained minimal.

The image quality function D and the image structural similarity function SSIM were utilized to comprehensively evaluate the reconstructed image quality. The function D represented the error between the pixel values of each unit in the reconstructed image before and after reconstruction, when compared to the model image pixel values. On the other hand, SSIM comprehensively compared the brightness, contrast, and structure of the reconstructed image with the model image. These two evaluation functions were combined and assessed comprehensively, defined as the function δ. As SSIM provided a more comprehensive evaluation compared to D, a weight of 0.4 was assigned to D, while a weight of 0.6 was assigned to (1‐SSIM).

### 2.5 Detection of Pork Quality by EIT System

Stainless‐steel electrodes were utilized across all detectors, with the detection parameters configured to an adjacent excitation pattern and an excitation current of 15 mA at a frequency of 10 kHz. Stainless‐steel alligator clips served as the interface to connect the hardware system with the electrode system, while sophisticated software was employed to regulate the injection of excitation currents and facilitate the collection of voltage data between adjacent electrodes on the boundary for subsequent analysis and imaging purposes.

1. For the experimentation, pork samples were sourced from the same anatomical region as the test specimens. Detectors with diameters of 5 cm and 10 cm were employed in the study. Two distinct measurement modalities were utilized: one involving direct contact with the surface of the pork, and the other entailing the embedding of electrodes into the pork samples. Under each experimental condition, 12 pork samples of differing quality were subjected to testing, with data collection performed six times for each individual sample to ensure robustness and reliability of the findings.
2. Impedance imaging tests were conducted on pork samples exhibiting varying degrees of freshness. Following each boundary voltage measurement, the samples were placed in a controlled environment chamber maintained at a constant temperature of 35°C and humidity level of 70%. This specific environmental setting was chosen to facilitate bacterial growth and accelerate the process of meat spoilage. The pork samples were allowed to undergo deterioration for durations of 0 h, 2 h, 4 h, 6 h, and 8 h, respectively. To ensure robust data collection, measurements were taken six times for each sample at each time point.

### 2.6 Sensory Evaluation of Pork Quality

#### 2.6.1 Establishment of Sensory Evaluation Panel

A total of 16 male and 16 female sensory assessors, aged between 20 and 35 years, were carefully selected for the study. These individuals possessed normal color vision, with no instances of color blindness or weakness. Furthermore, they were devoid of any cultural or religious taboos and did not harbor any aversion to pork. The chosen assessors underwent intensive training in sensory evaluation every two days over a period of two weeks. This rigorous training process ultimately led to the final selection of 15 male and 15 female assessors, who formed the sensory panel for the study. The training details were as follows:

1. distinguishing of sour, sweet, bitter, salty, and umami taste.
2. evaluation of pork samples with different ratio of muscle and fat.
3. be familiar with the experimental protocol.

Smoking and eating before each sensory evaluation were not allowed.

#### 2.6.2 Sensory Evaluation

In accordance with national and industry standards, relevant literature, consumer purchasing habits, and direct input from sensory assessors describing actual samples, a comprehensive set of attribute words was compiled. Subsequently, a panel of professional sensory assessors convened to engage in a thorough discussion and refinement of these attributes. This was followed by an additional refinement process conducted by the sensory assessors themselves through discussion. The final evaluation criteria were established through three rounds of iterative refinement, ensuring a robust and reliable framework. The refined attribute words, as derived from the sensory analysis, were then systematically compiled into a sensory evaluation form for further analysis and application.

During the formal experiment, the samples were randomly distributed to ensure unbiased evaluations. Each assessor then evaluated the attributes of each sample based on the sensory evaluation table. They recorded their evaluations for each attribute word systematically until they had completed the evaluation of all six samples. The pork quality was assessed using a scoring method, where the scales for each option were quantified and tallied to arrive at a final score, providing a comprehensive and quantitative evaluation of the samples.

### 2.7 Data Processing

#### 2.7.1 Hardware System Data

In the context of adjacent and opposite excitation patterns, a total of 208 and 192 voltage data points, respectively, could be measured. Specifically, for the 16 channels under consideration, each channel generates 13 and 12 voltage data points in these two excitation patterns. To assess the consistency across the channels, the voltage curves from all channels were amalgamated and presented collectively. The SNR and stability of the system were subsequently quantified using the voltage data processed through the following formula.

$$\mathrm{SNR}=10\log\frac{\sum_{n=1}^{N}V_{n}^{2}}{\sum_{n=1}^{1^{v}}{\left(V_{n}-V_{\text{mean}}\right)}^{2}}$$

$$\delta_{\max}=\frac{\left|V_{\max}-V_{\text{mean}}\right|}{V_{\text{mean}}}\times 100\%$$

*N* is the number of tests. *V*_*n*_ is the measured voltage value, *V*_mean_ is the average value of all measurements, *V*_max_ is the maximum value of the measured voltage, *δ*_max_ is the relative error in maximum voltage measurement of the system.

#### 2.7.2 DOE Experimental Data

1. Graph binarization

In this study, an analysis of the RGB channel characteristics of the image was conducted. Subsequently, the RGB values of the foreign object were extracted. The image was segmented, followed by binarization. All RGB channels of pixels within the target area were set to 0, thereby displaying them as black. Conversely, pixels in other areas, considered part of the background, had all their RGB channels set to 255.

2 Function δ

The image quality function *D*, pertaining to the model image *X* and the reconstructed image *Y*, was defined as follows:

$$D=\frac{\sum_{i=1}^{m}\sum_{j=1}^{m}\frac{\left|X\left(i，j\right)-Y\left(i，j\right)\right|}{255}}{M}$$

$$\text{SSIM}\left(X,Y\right)=\frac{4\mu_{x}\mu_{y}\sigma_{\mathrm{xy}}}{\left({\mu_{x}}^{2}+{\mu_{y}}^{2}\right)\left({\sigma_{x}}^{2}+{\sigma_{y}}^{2}\right)}$$

$$\delta =\left(1-\text{SSIM}\right)\times 0.6+D\times 0.4$$

*X* (*i*, *j*) is the pixel value of point (*i*, *j*) of model image, *Y* (*i*, *j*) is the pixel value of point (*i*, *j*) of reconstructed image, *M* is the total number of pixels, and m is the image resolution (pixels) in *X* and *Y* axes.

$\mu_{x}$, $\mu_{y}$, ${\sigma_{x}}^{2}$, ${\sigma_{y}}^{2}$, $\sigma_{\mathrm{xy}}$ represent the mean, variance, and covariance of pixel values of model image *X* and reconstructed image *Y*, respectively, and the calculation formula is as follows:

$$\mu_{x}=\frac{1}{m^{2}}\sum_{i=1}^{m}\sum_{j=1}^{m}X\left(i，j\right)$$

$$\mu_{y}=\frac{1}{m^{2}}\sum_{i=1}^{m}\sum_{j=1}^{m}Y\left(i，j\right)$$

$${\sigma_{x}}^{2}=\frac{1}{m^{2}}\sum_{i=1}^{m}\sum_{j=1}^{m}\left[X\left(i，j\right)-\mu_{x}\right]$$

$${\sigma_{y}}^{2}=\frac{1}{m^{2}}\sum_{i=1}^{m}\sum_{j=1}^{m}\left[Y\left(i，j\right)-\mu_{y}\right]$$

$$\sigma_{\mathrm{xy}}=\frac{1}{m^{2}}\sum_{i=1}^{m}\sum_{j=1}^{m}\left[X\left(i，j\right)-\mu_{x}\right]\left[Y\left(i，j\right)-\mu_{y}\right]$$

3 Analysis method

The data were examined for potential anomalies, ensuring that the observed pattern conformed to a normal distribution. The data were subjected to a standardized *T*‐test, and the obtained values were arranged in descending order to construct a Pareto effect diagram. Subsequently, residual analysis was performed to assess the quality of the model and its fit to the data. Based on the model judgment coefficient and the results of the residual analysis, the model was evaluated and determined, leading to the derivation of the regression equation. Thereafter, utilizing the “optimizing responder” function in Minitab, the response *δ* value was set to the minimum, and the conditional parameters were optimized to obtain the optimal parameters of the model.

#### 2.7.3 Sensory Data

During the processing of sensory data, it was necessary to eliminate abnormal values. Following the removal of these aberrant values, the average value and total score for each index were calculated. The ratio of the average value of each attribute to the total score of the average values constituted the weight value of the attribute. Through discussions among the sensory panel, five attribute words were ultimately determined: distribution of marbling, distribution of fat and lean, flesh color, fat color, and luster. The sensory score was then calculated according to the following formula.

$$\begin{array}{c} \text{Sensoy evaluation score}=0.11S_{\text{marbling}}+0.31S_{\mathrm{fat}\;\text{and lean}}\\ +0.28S_{\text{flesh color}}+0.13S_{\mathrm{fat}\;\text{color}}+0.17S_{\text{luster}} \end{array}$$

S_marbling_, S_fat and lean_, S_flesh color_, S_fat color_, and S_luster_ are the sensory scores of distribution of marbling, distribution of fat and lean, flesh color, fat color, and luster, respectively.

The sensory evaluation data of each sample were analyzed to identify significant differences, and a diagram was constructed to illustrate the significant disparities in sensory data among the attributes of each sample.

#### 2.7.4 Imaging Evaluation Data

The impedance information derived from imaging was employed as the detection variable for distinct pork samples, where the row vector denoted the samples and the column vector represented the variables. The impedance data pertaining to various samples were systematically organized into a data table for the purpose of conducting principal component analysis (PCA). Subsequently, by juxtaposing the principal component score maps of different samples within a two‐dimensional plane, a comparative analysis was performed to assess the discriminatory efficacy of each sample's impedance information within this plane.

## 3 Results And Discussion

### 3.1 EIT System Test Results

#### 3.1.1 Channel Consistency

Channel consistency result is shown in Figure 2. It was observed that the current density was higher in the region proximate to the excitation electrode, resulting in a “double U”‐shaped distribution of relative excitation pattern (shown in Figure 2a) and a “U”‐shaped distribution of adjacent excitation pattern (shown in Figure 2b), attributable to the elevated potential value. Furthermore, symmetrically distributed measured data were observed across each channel. In both adjacent and relative excitation scenarios, curves corresponding to high frequencies, when compared to low frequencies, and low currents, when compared to high currents, exhibited greater fluctuations and a lower degree of overlap.

Optimal channel consistency was achieved under 15 mA‐10 kHz excitation conditions, whereas it was suboptimal under 1.5 mA‐50 kHz excitation conditions. The measured data closely aligned with the theoretical analysis, indicating that the designed excitation measurement channel for this system exhibited a certain level of consistency. The voltage data displayed well‐defined curves, demonstrating good resolution and overall system performance.

#### 3.1.2 SNR And System Stability

The SNR and *δ*_max_ results obtained from each excitation condition are listed in Table 1. The SNR of the excitation current at a frequency of 10 kHz was significantly larger than that at 50 kHz, with both exceeding 60 dB. Furthermore, the stability of the excitation current at 10 kHz was considerably better than that at 50 kHz, with both being less than 0.5%. Under conditions without any foreign objects in the system, using an excitation current of 15 mA at 10 kHz, the *δ*_max_ values measured by the silver electrode, copper electrode, and stainless‐steel electrode were 81.12%, 11.23%, and 8.58%, respectively. When foreign objects were present in the system, the *δ*_max_ values generated by these three electrodes under the same excitation current were 3.94%, 3.53%, and 3.52%, respectively. The excitation condition of 15 mA at 10 kHz exhibited good system stability, which was consistent with the aforementioned conclusion.

**TABLE 1:** The SNR and*δ*_max_ of the system under different detection conditions.

Excitation Conditions		SNR	*δ*max			
		Fixed resistor	Fixed resistor	Silver electrode	Copper electrode	Stainless‐steel electrode
With foreign objects	1.5 mA‐10 kHz	64.9	0.1	123.10	64.19	119.62
	1.5 mA‐50 kHz	36.9	2.5	158.91	233.86	204.27
	15 mA‐10 kHz	62.7	0.2	81.12	11.23	8.58
	15 mA‐50 kHz	35.4	3.5	46.52	53.53	27.38
Without foreign objects	1.5 mA‐10 kHz	—	—	30.60	20.56	178.7
	1.5 mA‐50 kHz	—	—	102.72	90.86	27.35
	15 mA‐10 kHz	—	—	3.94	3.53	3.52
	15 mA‐50 kHz	—	—	21.61	26.00	110.67

#### 3.1.3 Foreign Object Imaging

**FIGURE 3:**
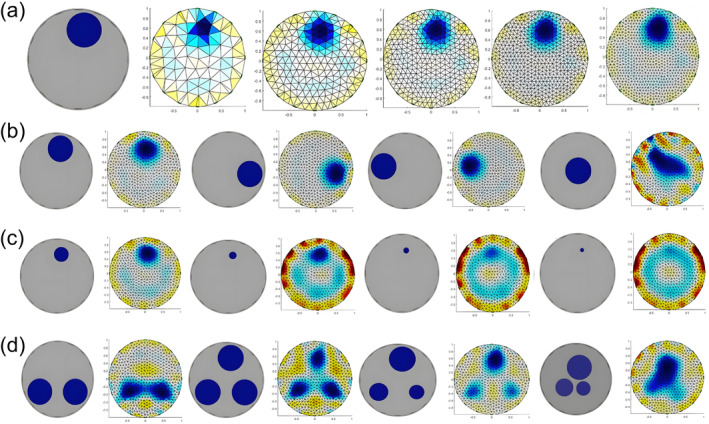
Image reconstruction evaluation results of different foreign objects: (a) diverse grid‐based imaging assessment, (b) imaging results of foreign objects situated between various electrodes, (c) imaging results of foreign objects with varying diameters, and (d) imaging results for varying quantities of foreign objects and differing diameters of these objects.

As depicted in Figure 3a, an augmentation in the number of finite elements led to a more precise division of the field, ultimately bringing the reconstructed image of the foreign object closer to reality and enhancing the resolution of the imaging. However, a sustained increase in the number of finite elements resulted in an exponential escalation of calculation time, potentially culminating in imaging failures within complex systems. After balancing various factors, this system opted for a finite element model comprising 1108 elements to ensure adequate resolution during the image reconstruction process.

**TABLE 2:** Sensory evaluation results of pork samples.

Sample name	Distribution of marbling	Distribution of fat and lean	Flesh color	Fat color	Luster	Total score
S1	9	15	19	15	15	73
S2	11	15	15	15	17	73
S3	10	14	14	14	18	70
S4	9	13	17	16	19	75
S5	11	14	19	14	16	74
S6	5	15	19	16	16	70

Figure 3b,c illustrates that when foreign objects were situated near the electrode, their images were notably clear and well‐positioned. Conversely, when these objects were positioned in the center of the field, the accuracy of their shape and position diminished, primarily attributable to the varying current densities. Larger foreign objects, exceeding 10 mm in size, produced clear and precise images. However, as the size of these objects decreased, the accuracy of positioning correspondingly declined. The system demonstrated the capability to detect foreign objects with a minimum diameter of 7.5 mm. As shown in Figure 3d, it accurately reconstructed images for varying numbers of foreign objects, particularly when they were located at the periphery. Nonetheless, in scenarios where there were numerous objects of similar size, the reconstructed image might exhibit slight distortions. When objects were positioned in the central region, they tended to blend, rendering it challenging to ascertain their exact count or shape.

### 3.2 DOE Results

**FIGURE 4:**
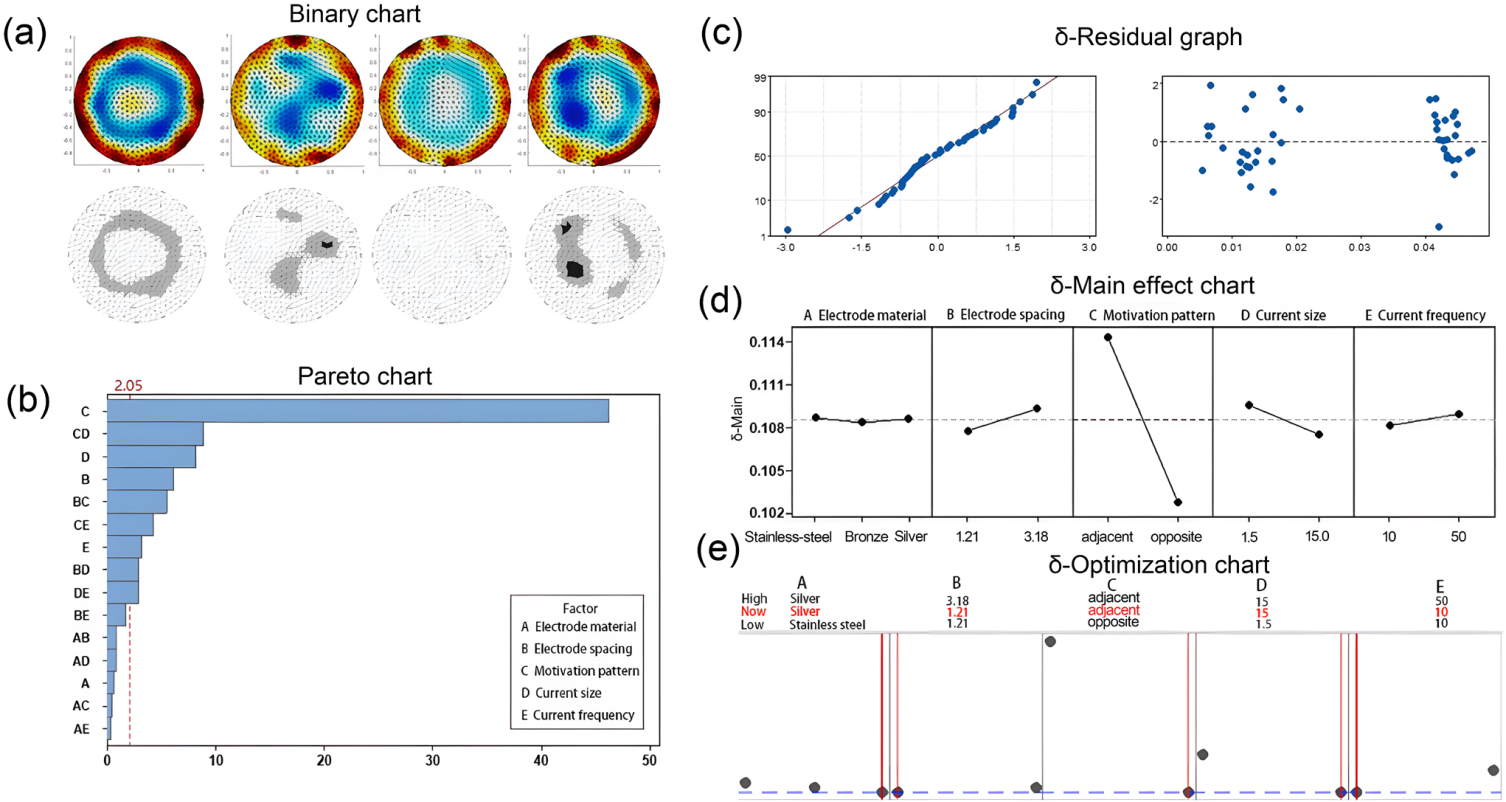
Results of DOE: (a) binary result, (b) Pareto result, (c) δ‐Residual result, (d) δ‐Main effect result, and (e) δ‐Optimization result.

The Pareto chart presented in Figure 4 elucidates that, at a significance level of ɑ = 0.05, factors surpassing the critical t‐value of 2.05 were deemed significant. The order of significance was observed to be C>C*E>D>C*D>E (“*” means interaction effect). Notably, the effect of factor E was lesser than the interaction effect of C and E, suggesting that the impact of excitation mode on image quality was constrained by the current frequency. The slope of the main effect plot served as an indicator of the significance of factors on δ. A steeper slope was indicative of a more pronounced effect. In this context, the excitation mode emerged as a strongly significant factor, whereas the electrode material exhibited insignificance.

The residual plot indicated a satisfactory fit of the model, with an R‐Sq value of 98.87% and an R‐Sq (adjusted) value of 98.03%, differing by a mere 0.84%. This minimal difference suggested that there was no necessity for model simplification, thus allowing the model to be utilized for parameter optimization. Based on the δ‐optimization plot, the optimal EIT parameters for the foreign body‐free model were determined to be: stainless‐steel electrode material, an electrode spacing of 1.21 cm, an adjacent excitation pattern, and a current of 15 mA at a frequency of 10 kHz.

Upon conducting three repetitions of the experiment under the optimized conditions, a fitted value of 0.0053 was obtained, which corresponded to a 95% confidence interval of 0.0022–0.0083 and a 95% prediction interval of 0.0003–0.0108. The average measurement of 0.005178 fell within these intervals, thereby confirming the credibility of the optimal parameters derived from the fitting process.

### 3.3 Pork Quality Evaluation

#### 3.3.1 Imaging Evaluation

**FIGURE 5:**
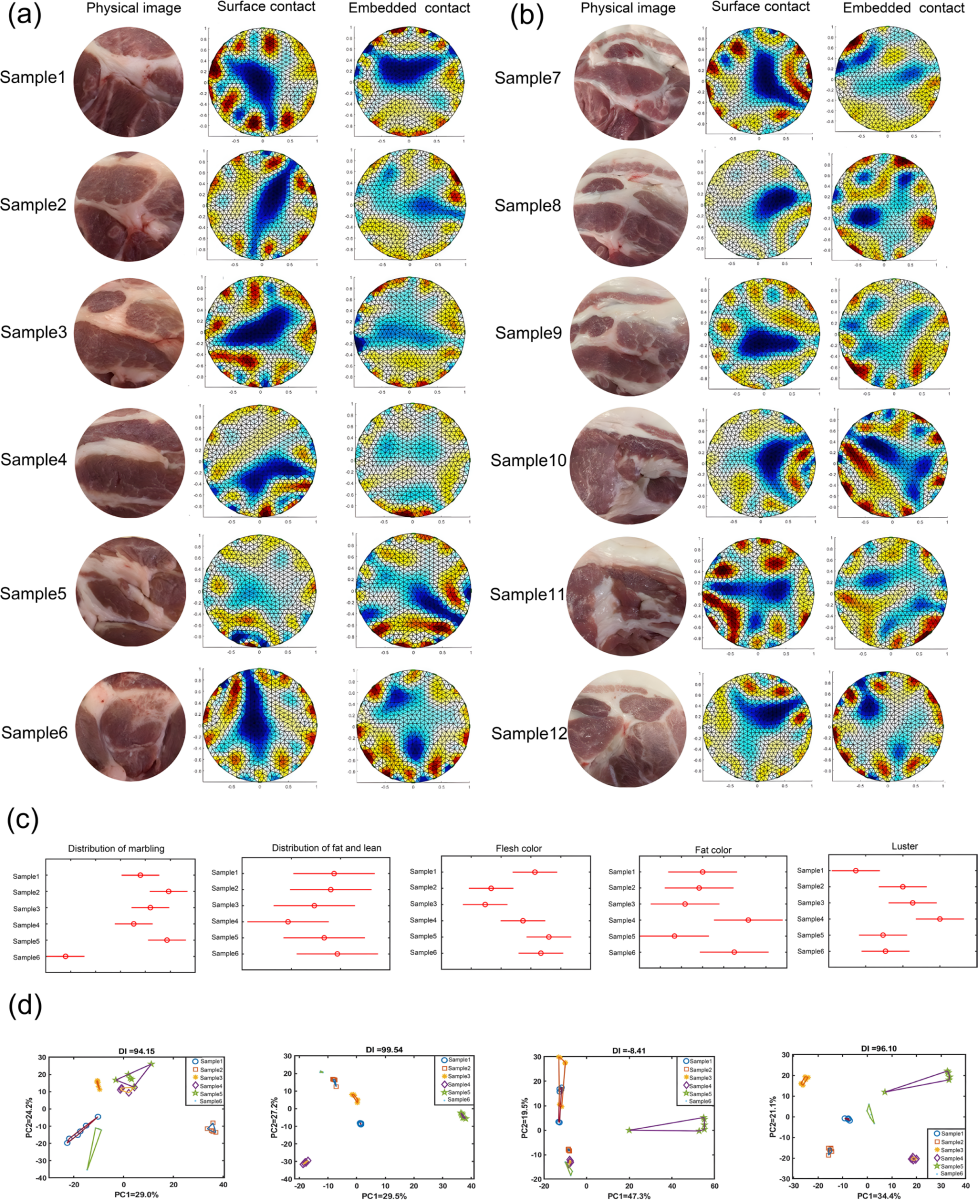
Pork quality classification results: (a) 5 cm of detector diameter under surface contact and embedded detection modes, (b) 5 cm of detector diameter under surface contact and embedded detection modes, (c) sensory evaluation significance analysis chart of five attributes, and (d) PCA results for pork samples with different quality.

The sensor imaging outcomes for various pork samples are presented in Figure 5a,b. Specifically, Figure 5a illustrates the results obtained from surface contact and embedded measurements using a detector with a diameter of 5 cm, whereas Figure 5b exhibits the outcomes derived from a detector with a diameter of 10 cm. In these figures, the blue regions denote the fatty portions, and the remaining areas represent the muscular components. As evident from the figures, sensors with different diameters were capable of reconstructing images that accurately portrayed the intrinsic fat and muscle structures of the pork samples across different measurement modalities. Nonetheless, it was discernible that the image results attained by the detector with a 5 cm diameter in the embedded measurement mode exhibited greater congruity with the actual sample quality.

#### 3.3.2 Quality Classification

Figure 5c showed the results of sensory eavluation scores in Table 2. It could be seen that the six samples had significant differences in sensory quality. Furthermore, the results of the principal component analysis for the 5 cm and 10 cm diameter detectors in both surface contact and embedded detection modes are depicted in Figure 5d. It was evident from the figure that the principal component analysis yielded optimal results when utilizing a 5 cm diameter detector in embedded detection mode. This finding was consistent with the image reconstruction outcomes presented in section 3.3.1. Consequently, for subsequent pork sample detection, the use of a 5 cm diameter detector in embedded detection mode is recommended.

#### 3.3.3 Freshness Discrimination

**FIGURE 6:**
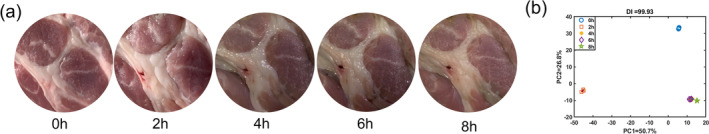
Freshness discrimination result.

Five distinct pork samples were subjected to varying periods of accelerated aging under optimized detection conditions prior to being analyzed through principal component analysis (PCA). The outcomes are depicted in Figure 6, where clear differentiation among these samples into three categories—0 h, 2 h, and 4‐8 h—is evident. Sensory evaluation, along with physical and chemical analyses, revealed that after a two‐hour period of accelerated aging, subtle degradation was observed in terms of alterations in moisture content, pH level, as well as meat texture. Subsequently, during an extended acceleration phase lasting between four to eight hours, there was a significant decline in pork quality compared to its initial state at both the 0‐h mark and after two hours. Henceforth, this established the viability of this testing methodology for assessing pork freshness.

## 4 Conclusions

This study employed a self‐constructed electrochemical impedance detection device and harnessed DOE technology to ascertain the optimal detection parameters, thereby establishing a methodology for image‐based analysis of meat sensory quality. Furthermore, principal component analysis was applied to the impedance data, based on the sensory evaluation results, enabling not only the differentiation of attribute variances among meat samples with similar overall sensory quality but also the identification of freshness disparities, achieving a discrimination accuracy exceeding 99%. Consequently, the developed detection method can be effectively utilized for the quality assessment and differentiation of pork and other meat samples.

## Author Contributions

**Yuezhong Mao:** data curation (equal), funding acquisition (equal), investigation (supporting), methodology (lead), writing – original draft (lead). **Chuanyong Yan:** data curation (lead), formal analysis (equal), investigation (equal), software (supporting), writing – original draft (supporting). **Xiaotong Liu:** data curation (supporting), formal analysis (lead), investigation (lead), resources (equal), software (equal). **Shuangni Shi:** investigation (supporting), methodology (supporting), project administration (supporting), visualization (supporting). **Yumei Qin:** project administration (supporting), resources (supporting), supervision (equal), writing – review and editing (supporting). **Shiyi Tian:** funding acquisition (equal), methodology (equal), supervision (lead), writing – review and editing (lead).

## Conflicts of Interest

The authors declare no conflicts of interest.

## Data Availability

The data that support the findings of this study are available from the corresponding author upon reasonable request.
